# Metabolites of bovine-associated non-*aureus* staphylococci influence expression of *Staphylococcus aureus agr*-related genes in vitro

**DOI:** 10.1186/s13567-021-00933-x

**Published:** 2021-04-29

**Authors:** Bruno Toledo-Silva, Fernando Nogueira de Souza, Sofie Piepers, Kristien Mertens, Freddy Haesebrouck, Sarne De Vliegher

**Affiliations:** 1 M-Team & Mastitis and Milk Quality Research Unit, Department of Reproduction, Obstetrics and Herd Health, Faculty of Veterinary Medicine, Ghent University, Merelbeke, Belgium; 2 Veterinary Clinical Immunology Research Group, Department of Clinical Science, Faculty of Veterinary Medicine and Animal Sciences, University of São Paulo, São Paulo, Brazil; 3 Postgraduate Program in Animal Science, Department of Veterinary Medicine, Federal University of Paraiba, Areia, Brazil; 4 Department of Pathology, Bacteriology and Avian Diseases, Faculty of Veterinary Medicine, Ghent University, Merelbeke, Belgium

**Keywords:** Coagulase-negative staphylococci, *Staphylococcus aureus*, *agr*, Quorum sensing, Mastitis

## Abstract

Communications via quorum sensing (QS) between non-*aureus* staphylococci (NAS) and *Staphylococcus (S.) aureus* in the bovine mammary gland remains largely unexplored. We determined whether 34 *S. chromogenes*, 11 *S. epidermidis*, and 14 *S. simulans* isolates originating from bovine milk samples and teat apices were able to regulate the QS of *S. aureus*, and if so, how in vitro growth inhibition of *S. aureus* by NAS, or NAS metabolites, or NAS cells themselves play a role in this process. In co-culture with *S. aureus* we observed that these 3 NAS species in general downregulated the expression of *rnaIII*, the effector molecule of the QS system, but this effect was more pronounced in *S. chromogenes* and *S. simulans* isolates than in *S. epidermidis* isolates. In vitro growth inhibition of *S. aureus* by NAS resulted in a small underestimation of the downregulating effect of NAS on *rnaIII* expression of *S. aureus*. Additionally, the culture supernatant of these NAS isolates and supernatant treated with proteinase K expressed greater regulatory activity over *S. aureus* virulence genes *rnaIII, hla*, and *spa* than washed NAS cells suspended in sterile water. These microbial interactions may influence *S. aureus* virulence and pathogenesis within the host. Isolation and identification of NAS metabolites affecting the QS system of *S. aureus* might help to develop alternative strategies for treatment and control of *S. aureus* mastitis.

**Supplementary Information:**

The online version contains supplementary material available at 10.1186/s13567-021-00933-x.

## Introduction

Bovine mastitis, an inflammation of the bovine mammary gland, can be caused by more than 100 bacterial species and subspecies [1]. The disease remains a major challenge to the dairy industry due to reduced milk production and quality, and substantial treatment costs [2]. One of the most common causative agents is *Staphylococcus aureus* [3–5]. *Staphylococcus aureus* remains extremely important for dairy herds because of its pathogenicity, contagiousness, capability to persist in the mammary gland, colonization of skin or mucosal epithelia, and poor cure rates when causing intramammary infections with the currently available therapies [3, 5].

The collective behaviors of bacteria are largely controlled by cell–cell communication or quorum sensing (QS), in response to changes in the population density and species composition of the adjacent community [6]. Quorum sensing via the accessory gene regulator (*agr*) system plays a significant role in the pathogenesis of staphylococci, especially *S. aureus* [7]. The *agr* system is composed of a 2-component signal transduction complex which in response to a secreted auto-inducing peptide (AIP) stimulates the expression of a regulatory RNA designated *rnaIII*, the effector molecule of the *agr* system [8]. The accumulation of AIP to a critical threshold drives the transcription of *rnaIII* which, as a reciprocal effect*,* results in upregulated expression of exoproteins, including *hla* encoding α-hemolysin, and downregulation of surface-associated proteins, such as protein A encoded by the *spa* gene [9, 10]. In addition to its regulation of the genes encoding individual virulence factors, QS coordinates activities including biofilm development, bioluminescence, bacterial competence, and virulence [11]. Therefore, QS has been the focus of many studies taking into account the potential advantages of using certain compounds for combating pathogens [12, 13].

The coagulase-negative staphylococci, now more often called the non-*aureus* staphylococci (NAS), have become the most common bacteria isolated from bovine milk samples [14–16]. The role of NAS for bovine udder health is under scrutiny, with recent work discussing a wide diversity between species and even strains in epidemiology, ecology, virulence, and host-interaction [17–21]. Previous studies reported NAS as a relevant cause of bovine mastitis [17, 22, 23], while others reported only a slight increase of the milk somatic cell count (SCC) as an indicator of mammary gland inflammation [24, 25] and no impact on milk yield, despite the elevated SCC [26]. A protective effect of NAS intramammary infections or NAS teat apex colonization against intramammary infections caused by major pathogens has also been reported [27–29]. Despite the literature supporting the predominance of NAS in the bovine mammary gland, the variation in the findings of research studies with regard to the role of the different bovine-associated NAS species originating from different habitats (e.g. milk, teat apex) needs further clarification.

*agr*-mediated interactions between NAS and *S. aureus* colonizing the same host niche have been recently suggested [30]. Some studies reported on the presence of potential NAS secreted compounds that might be responsible for the suppression of the *S. aureus* QS system, negatively affecting the ability of *S. aureus* to produce toxins [12, 31, 32]. However, to date, the interaction between *S. aureus* and NAS isolated from the bovine mammary gland or from bovine teat apices remains widely unstudied, except for one recent study [33]. Better insights in the interplay of NAS-*S. aureus* might be essential to understand the bacterial colonization process in the bovine mammary gland and to target *S. aureus* mastitis.

Based on the evidence that bacterial communications play an important role in niche generation and competition, we hypothesize that interactions of bovine NAS with *S. aureus* regulate the virulence of the latter in the bovine mammary gland. In order to investigate the hypothesis, we first investigated if bovine NAS isolates affect the *agr* QS of *S. aureus* when sharing the same niche, accounting for potential in vitro growth inhibition of *S. aureus* by NAS. Second, we examined whether metabolites produced and secreted by bovine NAS or NAS cells themselves influence the expression of *S. aureus* virulence factors controlled by the *agr* quorum sensing system.

## Materials and methods

### General study design

First, the in vitro growth inhibition of *S. aureus* by NAS was evaluated in order to reveal potential bactericidal effects of bovine NAS isolates (*n* = 59) belonging to three different species [*S. chromogenes* (*n* = 34)*, S. epidermidis* (*n* = 11)*,* and *S. simulans* (*n* = 14)] and originating from two different habitats, milk (45 isolates) and teat apices (14 isolates) of primiparous and multiparous cows (in vitro growth inhibition assay). Next, it was studied whether NAS can act as inhibitors of *S. aureus agr* by measuring the β-Galactosidase activity of *rnaIII::lacZ* (β-Galactosidase liquid assay), taking into account the in vitro growth inhibition results. Last, it was examined whether active substances produced by NAS or NAS cells themselves influence the expression of *S. aureus* QS-related genes (β-Galactosidase plate assay).

### Bacterial isolates

Non-*aureus* staphylococci isolates were obtained from our repository. The isolates were previously assigned to a species by matrix-assisted laser desorption/ionization-time of flight (MALDI-ToF) mass spectrometry analysis. In short, protein fingerprints of the isolates were compared with the commercial databank of bovine reference spectra (Bruker Daltonics), microbial spectra provided by Cameron et al. [34], and additional microbial spectra of field isolates from our lab covering 4 additional species (*S. jettensis*, *S. lentus*, *S. rostri*, and *S. saprophyticus*). The 59 NAS isolates were selected representing the three most prevalent species in milk samples and on teat apices of dairy cows and heifers [16, 35]: 45 from milk–*S. chromogenes* (*n* = 28), *S. epidermidis* (*n* = 7), and *S. simulans* (*n* = 10), and 14 from teat apices (TA)–*S. chromogenes* (*n* = 6), *S. epidermidis* (*n* = 4), and *S. simulans* (*n* = 4) (see Additional file 1). The *S. aureus* isolates used in this study and their references are listed in Additional file 1 as well.

Unless otherwise stated, bacteria were grown in Tryptic Soy Broth (TSB) for 16–24 h at 37 °C.

### In vitro growth inhibition assay

To evaluate potential growth inhibition of *S. aureus* by NAS, the cross-streaking method was used [27].

First, bacterial cultures of the 59 NAS isolates were adjusted to 0.5 McFarland turbidity standard, and inoculated as a center-streak (width of 5 mm) on Columbia Sheep Blood Agar Petri-dishes for 24 h at 37 °C. The agars were circularly loosened and then turned upside down and 0.5 McFarland standard suspensions of the *S. aureus* strain 8325–4 (see Additional file 1) was swabbed to achieve full coverage. The presence of a growth inhibition zone (measured in mm) within and adjacent to the center-streak was investigated after another 24 h of incubation for each NAS-*S. aureus* combination. As part of the set of 59 NAS isolates, the isolate SC29–“*S. chromogenes* TA”, a characterized inhibitor—*S. chromogenes* C2 in De Vliegher et al. [27]- was used as a positive control. A zone of total growth inhibition of *S. aureus* strain 8325–4 (TGI) was declared when no colonies were observed, whereas a zone of partial growth inhibition zone (PGI) was declared when smaller and/or less colonies of *S. aureus* strain 8325–4 were observed. If the same size/numbers of colonies of *S. aureus* strain 8325–4 were present as on the positive control plate (*S. aureus* 8325–4 alone), the zone was declared as no growth inhibition (NGI).

The experiments were performed in triplicate on three different days and results were averaged over the replicates. The distribution (%) of NAS inhibiting the growth of *S. aureus* 8325–4 was compared among the three different NAS species (*S. chromogenes*, *S. epidermidis*, *S. simulans*) and between the two different NAS habitats (milk and TA).

### β-Galactosidase liquid assay

This assay aimed to verify *S. aureus agr* inhibitory activity by bovine NAS when sharing the same niche environment. The overnight cultures of the SH101F7 *S. aureus rnaIII* reporter isolate [12] (see Additional file 1) and the 59 abovementioned NAS isolates were diluted 100 × in 15 mL of TSB, and allowed to reach an OD_600_ of 0.5. Each isolate was adjusted to an OD_600_ of 0.1 in TSB, and added to the same tube at a ratio of 1:1. From each culture, 1 mL was taken hourly (for 4 h) and centrifuged for 3 min at 7.2 *g* and 4 °C. The supernatants were removed after centrifugation, and the pellets recovered in 1 mL of TRIS 50 mM, pH 8 and 3 µL of lysostaphin. The mix was incubated at 37 °C for 30 min to allow cell lysis. Z-buffer (400 µL) was then added to each sample which was further incubated for 5 min at 28 °C. Lastly, 100 µL of ONPG (ortho-Nitrophenyl-β-galactosidase) (4 mg/mL) was added to the mix, and the time necessary for the solution to turn yellow was controlled.

Each assay was performed in triplicate on three different days. The absorbance at 420 and 550 nm for each sample was measured. The activity was calculated in Miller units as described by Miller [36]. It was analysed whether the *rnaIII* expression of *S. aureus* differed between the three different NAS species (*S. chromogenes*, *S. epidermidis*, *S. simulans*), between NAS originating from the two different habitats (milk and TA), and between NAS isolates based on their in vitro growth inhibition of the abovementioned *S. aureus* strain 8325–4 (no growth inhibition, partial inhibition and total inhibition as determined with the in vitro growth inhibition assay). *Staphylococcus schleiferi* 2898 served as positive control [12] and *rnaIII* expression of *S. aureus* in the absence of NAS served as negative control.

### β-Galactosidase plate assay

To determine whether active substances produced and secreted by NAS and NAS cells themselves have an effect on the expression of *S. aureus* virulence factors controlled by the *agr* quorum sensing system (QS) the β-Galactosidase plate assay was performed [37].

First, supernatants and bacterial cell suspensions were obtained from cultures of the 59 NAS isolates. Briefly, 16 h cultures of NAS isolates in TSB were centrifuged at 3000 *g* for 1 h [38] and the supernatants were carefully collected and filtered through 0.4 μm pore size filters. The pellets were washed twice with sterile water and suspended in 10 mL of sterile water, in order to obtain fresh NAS cell suspensions (CS). For each isolate, part of the filtered culture supernatant was left untreated (SP) and the other part was treated with proteinase K (SPK) at a concentration of 50 μg mL^−1^ for 1 h at 37 °C in a reaction mixture containing 0.5% SDS, 0.01 M Tris and 0.005 M EDTA. Following treatment, the enzyme was inactivated by adding phenylmethylsulfonyl fluoride. As an internal control, the supernatant of *S. aureus* 8325–4 (AIP-I; Additional file 1) was used to induce *agr*.

**Figure 1:**
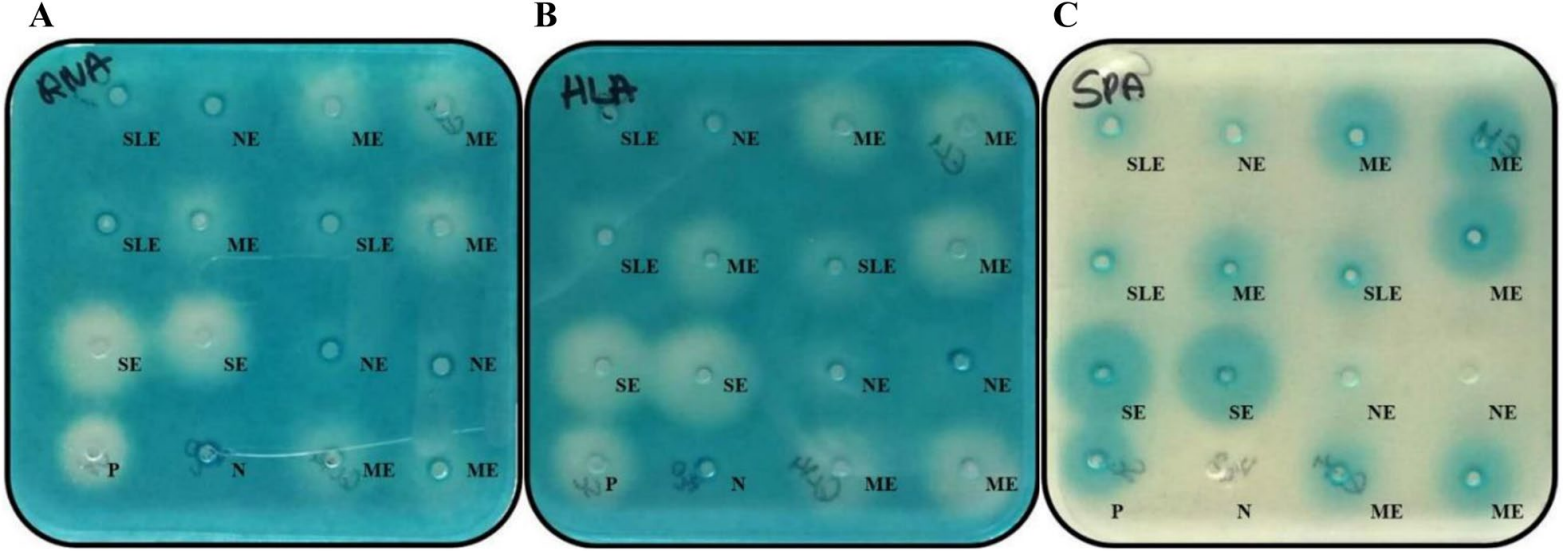
**Example of effect of non-aureus staphylococci isolates on gene expression of *Staphylococcus aureus*.** Tryptone soy agar plates (with erythromycin and X-gal) containing **(A)** the *rnaIII::lacZ* (SH101F7; Ery^r^), **(B)** the *hla::lacZ* (PC322; Ery^r^), or **(C)** the *spa::lacZ* (PC203; Ery^r^) reporter strain of *Staphylococcus aureus* were exposed to 20 μL of either Supernatant (SP), Supernatant + Proteinase K (SPK), or Cell suspension (CS) obtained from overnight cultures of *Staphylococcus chromogenes*, *Staphylococcus epidermidis*, and *Staphylococcus simulans*, respectively. *Staphylococcus schleiferi* (strain 2898) [12] and H_2_O were used as positive control (P) and negative control (N), respectively. Halos around the wells appeared between 12 and 36 h of incubation at 37 °C, and the diameter (measured in mm) was classified as NE: no effect (≤ 10 mm), SLE: slight effect (11–15 mm), ME: moderate effect (16–20 mm), and SE: severe effect (≥ 25 mm) on gene expression (upregulation for *rnaIII* and *hla*, and downregulation of *spa*). This figure is representative for all β-Galactosidase plate assays.

Then, three reporter strains of *S. aureus* carrying *lacZ* fused to central virulence genes (*hla, rnaIII*, and *spa*) encoding resistance to erythromycin (see Additional file 1) were grown for 16 h, and 2 mL of 10^3^-diluted cultures were placed in Greiner plates to which 50 mL of tryptone soy agar (TSA) (~40 °C) containing 150 µg/mL of 5-bromo-4-chloro-3-indolyl-D-galactopyranoside (X-Gal). In order to maintain selective pressure for the plasmids, 5 µg/mL of erythromycin was added to the agar. The plates were subsequently left to solidify, and wells were shaped manually with a sterile sharp iron drill (4 mm). Aliquots of 20 μL of SP, SPK, CS, and controls were added to plates containing the different reporter strains. The incubation time varied between the different reporter strains. Therefore, the plates were incubated at 37 °C for 9 to 36 h until a blue color appeared on the plates (Figure 1). The presence of a halo zone around the well indicated the regulation of the virulence genes (*rnaIII*, *hla* and *spa*) with the degree of the effect depending on the diameter of the halo zone (measured in mm) [12] and stratified as exhibiting no effect (≤ 10 mm), a slight effect (11–15 mm), a moderate effect (16–20 mm), or a severe effect (≥ 25 mm) on gene expression.

All experiments were performed in triplicate on three different days and results were averaged over replicates. The distribution (%) of NAS isolates affecting *S. aureus* virulence gene expression was compared between the three different NAS species (*S. chromogenes*, *S. epidermidis*, *S. simulans*), between the NAS belonging to the two different habitats (milk and TA) and between the three different culture preparations (SP, SPK and CS). The SP, SPK and CS of *S. schleiferi* 2898 [12] were used as positive controls, whereas H_2_0 was used as negative control.

## Statistical analyses

### β-Galactosidase liquid

The expression of the *rnaIII* gene of *S. aureus* (outcome variable) during co-culture with different NAS species (predictor variable of main interest) was studied using linear mixed models (PROC MIXED; SAS version 9.4; SAS Institute Inc., Cary, NC, USA) taking into account the repeated measurements. A natural logarithmic transformation of *rnaIII* expression values (Ln*RnaIII*) was performed to obtain a normal distribution.

First, a linear mixed model was fit with staphylococcal species [5 levels: *S. aureus* only (negative control)*, S. aureus* + *S. schleiferi* (positive control), *S. aureus* + *S. chromogenes, S. aureus* + *S. epidermidis*, and *S. aureus* + *S. simulans*] as a categorical predictor variable of main interest to compare NAS with the positive and negative controls, respectively. Time of measurement (4 levels: 1 h, 2 h, 3 h, and 4 h) and the interaction term between staphylococcal species and time of measurement were included as additional categorical predictor variables. Isolate was included as random effect to account for the three replicates per isolate and replicate was included as repeated effect to account for the 4 repeated measurements per replicate.

Second, a linear mixed model was fit with NAS species [3 levels: *S. aureus* + *S. chromogenes, S. aureus* + *S. epidermidis*, and *S. aureus* + *S. simulans*], the habitat of the NAS isolates (2 levels: milk and TA) and the in vitro growth inhibition of *S. aureus* by NAS (3 levels: total, partial, or no growth inhibition; see before) as categorical predictor variables and all possible two-way interactions. Isolate was included as random effect to account for the three replicates per isolate and replicate was included as repeated effect to account for the 4 repeated measurements per replicate. Non-significant variables (*p* > 0.05) were omitted from the full model using a backward stepwise approach. The goodness-of-fit measures included −2 × log-likelihood, the Akaike information criterion, and the Bayesian information criterion. Residuals were evaluated graphically and plotted against the predicted values. A Bonferroni’s correction was used to correct for multiple comparisons. Significance was assessed at *p* ≤ 0.05.

### β-Galactosidase plate assay

The Fisher’s exact test was used to determine whether the distribution of effects (no effect, slight effect, moderate effect, or severe effect on the expression of *rnaIII, hla,* and *spa* genes) differed (1) between NAS isolates belonging to the three different species (3 levels: *S. chromogenes*, *S. epidermidis*, and *S. simulans*), or (2) between NAS isolates originating from the two different habitats (2 levels: milk or teat apices), or (3) between the different culture preparations (3 levels: SP, SPK, CS). Statistical analyses were performed using SPSS v.26.0 (IBM Corp., Armonk, NY, USA) and *p* ≤ 0.05 was considered significant.

## Results

### In vitro growth inhibition

The patterns of the in vitro growth inhibition of *S. aureus* 8325–4 by the NAS isolates are presented in Table 1. Fifty NAS isolates out of 59 (84.7%) were able to inhibit the growth of *S. aureus* at least partially. Total growth inhibition was observed only by one *S. simulans* isolate from a TA and by the positive control strain *S. chromogenes* SC29—TA, also part of the set of 59 NAS isolates.

**Table 1:** **In vitro growth inhibition of** ***Staphylococcus aureus*** **8325–4** [44]** by bovine non-aureus staphylococci (NAS) isolates from bovine milk and teat apices (TA) [no. (%)]**

NAS species (no.)^1^	Habitat [no. (%)]		Total (59)
	Milk (45)	TA (14)	
All (59)			
Total growth inhibition	0	2 (14.3)	2 (3.4)
Partial growth inhibition	40 (88.9)	10 (71.4)	50 (84.7)
No growth inhibition	5 (11.1)	2 (14.3)	7 (11.9)
*Staphylococcus chromogenes* (34)			
Total growth inhibition	0	1 (16.7)	1 (2.9)
Partial growth inhibition	28 (100)	5 (83.3)	33 (97.1)
No growth inhibition	0	0	0
*Staphylococcus epidermidis* (11)			
Total growth inhibition	0	0	0
Partial growth inhibition	2 (28.6)	3 (75.0)	5 (45.4)
No growth inhibition	5 (71.4)	1 (25.0)	6 (54.6)
*Staphylococcus simulans* (14)			
Total growth inhibition	0	1 (25.0)	1 (7.1)
Partial growth inhibition	10 (100)	2 (50.0)	12 (85.8)
No growth inhibition	0	1 (25.0)	1 (7.1)

^1^Number of isolates.

### β-Galactosidase liquid assay

#### Comparison with the controls

**Figure 2:**
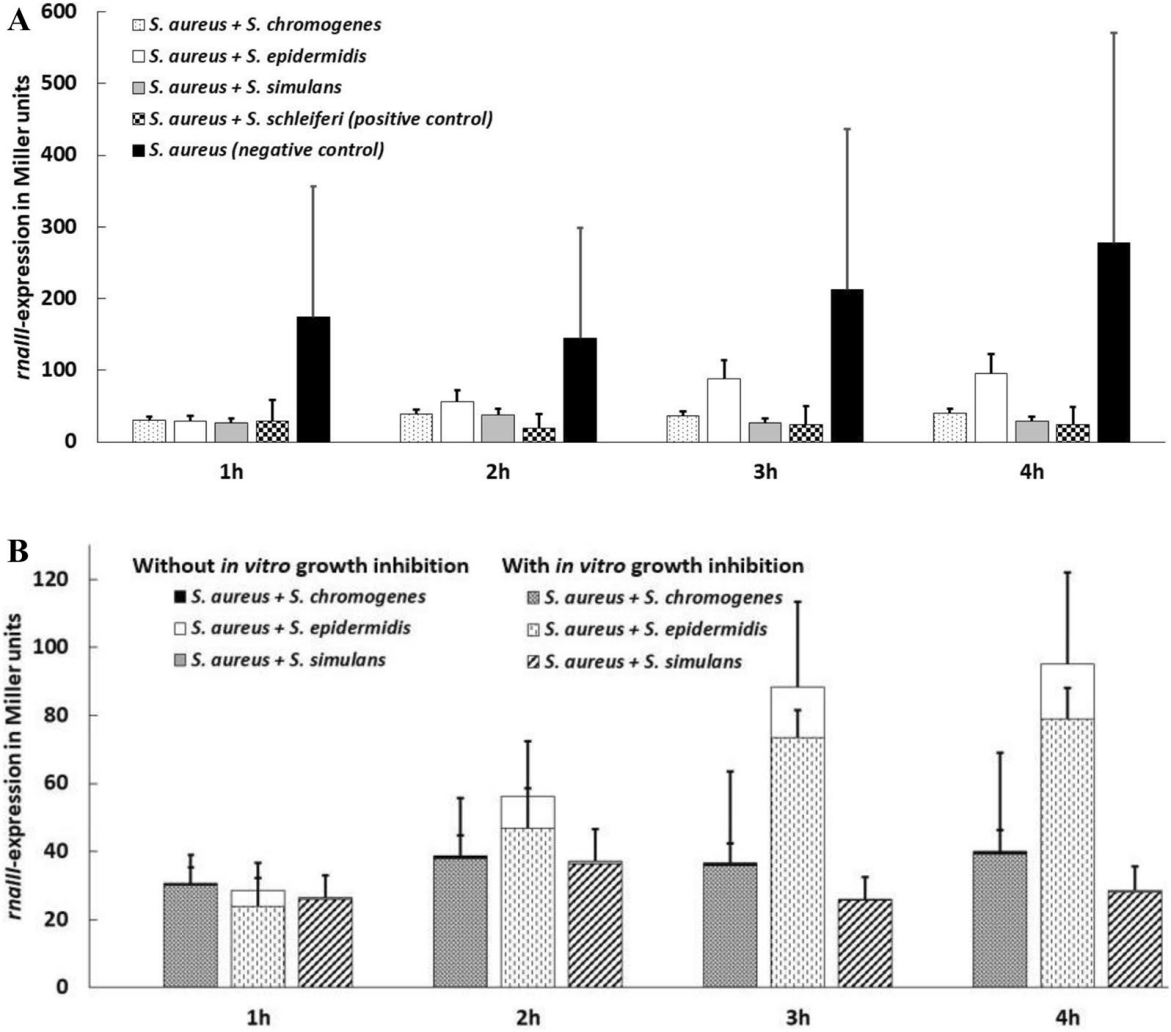
**β-Galactosidase liquid assay: effect of non-aureus staphylococci (NAS) on agr induction of** ***Staphylococcus aureus*** **over time. (A)** The β-galactosidase activity of *Staphylococcus aureus rnaIII*::*lacZ* reporter strain SH101F7 was monitored for 4 h growing alone (negative control) in TSB, or in co-culture (1:1) with *Staphylococcus chromogenes* (*n* = 34), *Staphylococcus epidermidis* (*n* = 11), *Staphylococcus simulans* (*n* = 14), and *Staphylococcus schleiferi* strain 2898 (positive control). **(B)** The *rnaIII* expression of NAS isolates affected (hatched bars) or not (solid bars) by NAS in vitro growth inhibition of *S. aureus* [27]. Each bar represents the least square means of three biological replicates and the error bars represent the standard deviation.

The *rnaIII* expression increased significantly over time [*p* = 0.0453; Least Square Means (LSM) increased from 40.95 to 58.95 back-transformed Miller units] and differed between staphylococcal species (*p* < 0.0001); yet the evolution of the expression over time significantly differed between staphylococcal species (*p* < 0.0001; Table 2 and Figure 2A). *Staphylococcus chromogenes* (Bonferroni corrected *p* = 0.0015; LSM = 36.2) and *S. simulans* (Bonferroni corrected *p* = 0.0004; LSM = 29.22) reduced *rnaIII* expression significantly more than the negative control (*S. aureus* only; LSM = 196.5) whereas this was not true for *S. epidermidis* (Bonferroni corrected *p* = 0.082; LSM = 60.63). The three species did not differ in the reduction of the *rnaIII* expression compared with *S. schleiferi* (positive control; LSM = 23.72) based on Bonferroni corrected *p*-values.

**Table 2:** **Final linear mixed effect model for the β-galactosidase liquid assay for** ***rnaIII* gene expression including the positive and negative controls**

Predictor variables	β^1^	SE^2^	LSM^3^	*P* value^4^
Intercept	2.24	0.20	–	
Time of measurement				0.0453
1 h	Referent	–	40.95	
2 h	−0.08	0.13	46.83	
3 h	0.09	0.16	53.35	
4 h	0.20	0.19	58.95	
Staphylococcal species^5^				< 0.0001
*S. aureus* (negative control)	Referent	–	196.52	
*S. aureus* + *S. schleiferi* (positive control)	−0.78	0.28	23.72	
*S. aureus* + *S. chromogenes*	−0.76	0.20	36.20	
*S. aureus* + *S. epidermidis*	−0.78	0.21	60.63	
*S. aureus* + *S. simulans*	−0.82	0.21	29.22	
Staphylococcal species x Time of measurement^6^				< 0.0001

^1^ Estimate.

^2^ Standard error.

^3^ Least square means after back transformation.

^4^ Overall *P* value for fixed effects.

^5^ *Staphylococcus aureus rnaIII*::*lacZ* reporter strain SH101F7 was used as negative control when growing alone and as positive control when in co-culture with *Staphylococcus schleiferi* strain 2898.

^6^ The interaction term is visualized in Figure 2A.

#### *Association with NAS species, NAS habitat and NAS *in vitro* growth inhibition*

The *rnaIII* expression increased significantly over time [*p* < 0.0001; LSM increased from 28.46 to 47.65] and differed between NAS species (*p* = 0.0002; lowest value for *S. simulans* (LSM = 29.22) and highest value for *S. epidermidis* (LSM = 60.63)]. *Staphylococcus epidermidis* (LSM = 60.63) reduced the *rnaIII* expression significantly less than *S. simulans* (Bonferroni corrected *p* = 0.0002; LSM = 29.22) and *S. chromogenes* (Bonferroni corrected *p* = 0.0021; LSM = 36.2). The evolution of the expression over time significantly differed between NAS species (*p* < 0.0001; Table 3 and Figure 2B). Neither the NAS habitat nor the NAS in vitro growth inhibition of *S. aureus* 8325–4 influenced the *rnaIII* expression significantly. However, forcing NAS in vitro growth inhibition into this final model in order to better understand whether the differences in effect between species was due to in vitro growth inhibition of *S. aureus* in the co-culture (Table 3, Figure 2B), the NAS species effect became slightly stronger (i.e. LSM values became smaller, indicating the reduction in *rnaIII* expression was more pronounced).

**Table 3:** **Final linear mixed regression model for the β-galactosidase liquid assay for** ***rnaIII* gene expression and final model corrected for in vitro growth inhibition of** ***Staphylococcus aureus*** **by bovine non-*aureus* staphylococci (NAS)**

Predictor variables	Final model				Final model corrected for in vitro growth inhibition			
	β^1^	SE^2^	LSM^3^	*p* value^4^	β	SE	LSM	*p* value
Intercept	1.42	0.05	–		1.53	0.10	–	
Time of measurement				< 0.0001				< 0.0001
1 h	Referent	–	28.46		Referent	–	26.45	
2 h	0.15	0.03	43.24		0.15	0.03	40.20	
3 h	−0.005	0.04	43.79		−0.005	0.04	40.70	
4 h	0.03	0.05	47.65		0.03	0.05	44.30	
NAS species				0.0002				0.0275
* S. aureus* + *S. chromogenes*	0.06	0.06	36.20		0.07	0.06	35.65	
* S. aureus* + *S. epidermidis*	0.04	0.08	60.63		–0.04	0.10	50.41	
* S. aureus* + *S. simulans*	Referent	–	29.22		Referent	–	28.67	
Habitat				NS^5^				
Milk	–	–	–	–				
Teat apex	–	–	–	–				
In vitro growth inhibition^6^				NS				(0.2957)^8^
None	–	–	–	–	Referent	–	48.76	
Partial	–	–	–	–	−0.11	0.10	38.14	
Total	–	–	–	–	−0.25	0.16	27.70	
NAS species × Time of measurement^7^				< 0.0001				< 0.0001
NAS species × Habitat	–	–	–	NS				–
NAS species × In vitro growth inhibition	–	–	–	NS				–
Habitat × In vitro growth inhibition	–	–	–	NS				–
Habitat × Time of measurement	–	–	–	NS				–

^1^Estimate.

^2^Standard error.

^3^Least square means after back transformation.

^4^Overall *P* value for fixed effects.

^5^Non-significant.

^6^See Materials and methods.

^7^The interaction term is visualized in Figure 2B.

^8^Forced into the model.

### β-Galactosidase plate assay

#### rnaIII

Downregulation of *rnaIII* differed between NAS species (*p* ≤ 0.001): 88% (29/33) of all culture preparations of the *S. epidermidis* isolates did not have an effect, whereas this was only 20.6% (21/102) and 16.7% (7/42) of the *S. chromogenes* and *S. simulans* isolates, respectively (highlighted by the bold type in Table 4). At the same time, downregulation of *S. aureus rnaIII* was similar between the habitats of the NAS isolates [*p* > 0.05; no effect in 32.6% (44/145) of all culture preparations of NAS isolates originating from milk and in 30.9% (13/42) of the isolates from TA]. Downregulation of *rnaIII* differed between the different culture preparations: there was no downregulation of *rnaIII* in 20.3 (12/59), 25.4 (15/59), and 50.8% (30/59) of the SP, SPK and CS, respectively (*p* ≤ 0.001).

**Table 4:** **Downregulation of the** ***rnaIII* gene activity of** ***Staphylococcus aureus* by three different culture preparations obtained from bovine non-*aureus* staphylococci (NAS) isolates from milk and teat apices (TA) [no. (%)]**

NAS species (no.)^1^	SP^2^ [no. (%)]			SPK [no. (%)]			CS [no. (%)]			All culture preparations [no. (%)]		
	Milk (45)	TA (14)	Total (59)	Milk (45)	TA (14)	Total (59)	Milk (45)	TA (14)	Total (59)	Milk (45)	TA (14)	Total (59)
All (59)												
Severe effect^3^	12 (26.7)	6 (42.8)	18 (30.5)	5 (11.1)	2 (14.3)	7 (11.9)	1 (2.2)	1 (7.1)	2 (3.4)	18 (13.4)	9 (21.4)	27 (15.2)
Moderate effect	24 (53.3)	2 (14.3)	26 (44.1)	26 (57.8)	7 (50.0)	33 (56.0)	2 (4.5)	4 (28.6)	6 (10.2)	52 (38.5)	13 (30.9)	65 (36.8)
Slight effect	1 (2.2)	2 (14.3)	3 (5.1)	3 (6.7)	1 (7.1)	4 (6.7)	17 (37.8)	4 (28.6)	21 (35.6)	21 (15.5)	7 (16.8)	28 (15.8)
No effect	8 (17.8)	4 (28.6)	**12 (20.3)**	11 (24.4)	4 (28.6)	**15 (25.4)**	25 (42.4)	5 (35.8)	**30 (50.8)**	**44 (32.6)**	**13 (30.9)**	57 (32.2)
*S. chromogenes* (34)												
Severe effect	2 (7.1)	2 (33.4)	4 (11.7)	2 (7.1)	0	2 (5.9)	1 (3.6)	1 (16.6)	2 (5.9)	5 (6.0)	3 (16.7)	8 (7.8)
Moderate effect	24 (85.7)	2 (33.4)	26 (76.5)	19 (67.9)	5 (83.4)	24 (70.6)	1 (3.6)	3 (50.0)	4 (11.7)	44 (52.4)	10 (55.5)	54 (53.0)
Slight effect	1 (3.6)	1 (16.6)	2 (5.9)	3 (10.7)	0	3 (8.8)	13 (46.4)	1 (16.7)	14 (41.2)	17 (20.2)	2 (11.1)	19 (18.6)
No effect	1 (3.6)	1 (16.6)	2 (5.9)	4 (14.3)	1 (16.6)	5 (14.7)	13 (46.4)	1 (16.7)	14 (41.2)	18 (21.4)	3 (16.7)	**21 (20.6)**
*S. epidermidis* (11)												
Severe effect	0	1 (25.0)	1 (9.1)	0	0	0	0	0	0	0	1 (8.3)	1 (3.0)
Moderate effect	0	0	0	0	1 (25.0)	1 (9.1)	1 (14.3)	1 (25.0)	2 (18.2)	1 (4.7)	2 (16.7)	3 (9.0)
Slight effect	0	0	0	0	0	0	0	0	0	0	0	0
No effect	7 (100)	3 (75.0)	10 (90.9)	7 (100)	3 (75.0)	10 (90.9)	6 (85.7)	3 (75.0)	9 (81.8)	20 (95.3)	9 (75.0)	**29 (88.0)**
*S. simulans* (14)												
Severe effect	10 (100)	3 (75.0)	13 (92.8)	3 (30.0)	2 (50.0)	5 (35.7)	0	0	0	13 (43.4)	5 (41.6)	18 (42.9)
Moderate effect	0	0	0	7 (70.0)	2 (50.0)	9 (64.3)	0	0	0	7 (23.3)	2 (16.7)	9 (21.4)
Slight effect	0	1 (25.0)	1 (7.2)	0	0	0	4 (40.0)	3 (75.0)	7 (50.0)	4 (13.3)	4 (33.4)	8 (19.0)
No effect	0	0	0	0	0	0	6 (60.0)	1 (25.0)	7 (50.0)	6 (20.0)	1 (8.3)	**7 (16.7)**

^1^ Culture preparations = SP–Supernatant; SPK–Supernatant + Proteinase K; CS–Cell suspension.

^2^ Number of isolates.

^3^ Regulation effect was rated according to the size of inhibition zone around the well (measured in mm) [12] and was classified as exhibiting no effect (≤ 10 mm), a slight effect (11–15 mm), a moderate effect (16–20 mm), and a severe effect (≥ 25 mm) on gene expression.

#### hla

No downregulation of *hla* was observed in 79% (26/33) of all culture preparations of the *S. epidermidis* isolates while, on the contrary, this was 22.5% (23/102) and 14.3% (6/42) of the *S. chromogenes* and *S. simulans* isolates, respectively (*p* ≤ 0.001) (highlighted by the bold type in Table 5). Conversely, the NAS habitat did not influence *hla* expression [*p* > 0.05; no effect in 31.1% (42/145) of all culture preparations of NAS originating from milk and in 30.9% (13/42) of the isolates from TA]. Moreover, downregulation of the *hla* gene differed between the culture preparations (*p* ≤ 0.001), since 18.7% (11/59) of the SP, 20.3% (12/59) of the SPK*,* and 54.2% (32/59) of the CS showed no downregulation.

**Table 5:** **Downregulation of the** ***hla* gene activity of** ***Staphylococcus aureus* by three different culture preparations obtained from bovine non-*aureus* staphylococci (NAS) isolates from milk and teat apices (TA)**

NAS species (no.)^1^	SP^2^ [no. (%)]			SPK [no. (%)]			CS [no. (%)]			All culture preparations [no. (%)]		
	Milk (45)	TA (14)	Total (59)	Milk (45)	TA (14)	Total (59)	Milk (45)	TA (14)	Total (59)	Milk (45)	TA (14)	Total (59)
All (59)												
Severe effect^3^	26 (57.8)	9 (64.3)	35 (59.3)	7 (15.6)	3 (21.4)	10 (17.0)	0	0	0	33 (24.5)	12 (28.5)	45 (25.4)
Moderate effect	11 (24.4)	1 (7.1)	12 (20.3)	27 (60.0)	7 (50.0)	34 (57.6)	2 (4.5)	0	2 (3.4)	40 (29.6)	8 (19.0)	48 (27.1)
Slight effect	1 (2.2)	0	1 (1.7)	3 (6.6)	0	3 (5.1)	16 (35.5)	9 (64.3)	25 (42.4)	20 (14.8)	9 (21.4)	29 (16.4)
No effect	7 (15.6)	4 (28.6)	**11 (18.7)**	8 (17.8)	4 (28.6)	**12 (20.3)**	27 (60.0)	5 (35.7)	**32 (54.2)**	**42 (31.1)**	**13 (30.9)**	55 (31.1)
*S. chromogenes* (34)												
Severe effect	16 (57.1)	4 (66.8)	20 (58.8)	3 (10.7)	1 (16.6)	4 (11.7)	0	0	0	19 (22.6)	5 (27.8)	24 (23.5)
Moderate effect	11 (39.3)	1 (16.6)	12 (35.3)	21 (75.0)	4 (66.8)	25 (73.6)	1 (3.6)	0	1 (2.9)	33 (39.3)	5 (27.8)	38 (37.3)
Slight effect	0	0	0	3 (10.7)	0	3 (8.8)	9 (32.1)	5 (83.4)	14 (41.2)	12 (14.3)	5 (27.8)	17 (16.7)
No effect	1 (3.6)	1 (16.6)	2 (5.9)	1 (3.6)	1 (16.6)	2 (5.9)	18 (64.3)	1 (16.7)	19 (55.9)	20 (23.8)	3 (16.6)	**23 (22.5)**
*S. epidermidis* (11)												
Severe effect	0	1 (25.0)	1 (9.1)	0	1 (25.0)	1 (9.1)	0	0	0	0	2 (16.7)	2 (6.0)
Moderate effect	0	0	0	0	0	0	1 (14.3)	0	1 (9.1)	1 (4.7)	0	1 (3.0)
Slight effect	1 (14.3)	0	1 (9.1)	0	0	0	2 (28.6)	1 (25.0)	3 (27.3)	3 (14.3)	1 (8.3)	4 (12.0)
No effect	6 (85.7)	3 (75.0)	9 (81.8)	7 (100)	3 (75.0)	10 (90.9)	4 (57.1)	3 (75.0)	7 (63.6)	17 (81.0)	9 (75.0)	**26 (79.0)**
*S. simulans* (14)												
Severe effect	10 (100)	4 (100)	14 (100)	4 (40.0)	1 (25.0)	5 (35.7)	0	0	0	14 (46.6)	5 (41.6)	19 (45.3)
Moderate effect	0	0	0	6 (60.0)	3 (75.0)	9 (64.3)	0	0	0	6 (20.0)	3 (25.0)	9 (21.4)
Slight effect	0	0	0	0	0	0	5 (50.0)	3 (75.0)	8 (57.2)	5 (16.7)	3 (25.0)	8 (19.0)
No effect	0	0	0	0	0	0	5 (50.0)	1 (25.0)	6 (42.8)	5 (16.7)	1 (8.3)	**6 (14.3)**

^1^ Number of isolates.

^2^Culture preparations = SP–Supernatant; SPK–Supernatant + Proteinase K; CS–Cell suspension.

^3^Regulation effect was rated according to the size of inhibition zone around the well (measured in mm) [12] and was classified as exhibiting no effect (≤ 10 mm), a slight effect (11–15 mm), a moderate effect (16–20 mm), and a severe effect (≥ 25 mm) on gene expression.

#### spa

Different effects on upregulation of the *spa* gene were also observed between NAS species (*p* ≤ 0.001): no effect was observed in 79% (26/33) of all culture preparations of the *S. epidermidis* isolates, whereas this was only 21.6% (22/102) of *S. chromogenes* isolates and 9.5% (4/42) of *S. simulans* isolates (highlighted by the bold type in Table 6). Upregulation of the *spa* gene was similar between NAS habitats (*p* > 0.05): upregulation of the gene was absent in 28.2% (38/145) of all culture preparations of NAS isolates originating from milk and in 33.3% (14/42) from TA. Regulation of *spa* gene expression depended on the culture preparations (*p* ≤ 0.001): no effect was present in 18.7 (11/59), 20.3 (12/59), and 49.1% (29/59) of the SP, SPK, and CS preparations, respectively.

**Table 6:** **Upregulation of the** ***spa* gene activity of** ***Staphylococcus aureus* by three different culture preparations obtained from bovine non-*aureus* staphylococci (NAS) isolates from milk and teat apices (TA)**

NAS species (no.)^1^	SP^2^ [no. (%)]			SPK [no. (%)]				CS [no. (%)]			All culture preparations [no. (%)]		
	Milk (45)	TA (14)	Total (59)	Milk (45)	TA (14)	Total (59)		Milk (45)	TA (14)	Total (59)	Milk (45)	TA (14)	Total (59)
All (59)													
Severe effect^3^	20 (44.4)	8 (57.1)	28 (47.4)	6 (13.4)	2 (14.3)	8 (13.6)		0	0	0	26 (19.2)	10 (23.8)	36 (20.3)
Moderate effect	17 (37.8)	2 (14.3)	19 (32.2)	11 (24.4)	8 (57.1)	19 (32.2)		4 (8.9)	1 (7.1)	5 (8.5)	32 (23.7)	11 (26.2)	43 (24.3)
Slight effect	1 (2.2)	0	1 (1.7)	20 (44.4)	0	20 (33.9)		18 (40.0)	7 (50.0)	25 (42.4)	39 (28.9)	7 (16.7)	46 (26.0)
No effect	7 (15.6)	4 (28.6)	**11 (18.7)**	8 (17.8)	4 (28.6)	**12 (20.3)**		23 (51.1)	6 (42.9)	**29 (49.1)**	**38 (28.2)**	**14 (33.3)**	52 (29.4)
*S. chromogenes* (34)													
Severe effect	11 (39.3)	4 (66.8)	15 (44.1)	2 (7.1)	0	2 (5.9)		0	0	0	13 (15.5)	4 (22.2)	17 (16.7)
Moderate effect	15 (53.6)	1 (16.6)	16 (47.1)	5 (17.9)	5 (83.4)	10 (29.4)		2 (7.1)	1 (16.6)	3 (8.8)	22 (26.2)	7 (38.9)	29 (28.4)
Slight effect	1 (3.6)	0	1 (2.9)	20 (71.4)	0	20 (58.8)		10 (35.7)	3 (50.0)	13 (38.2)	31 (36.9)	3 (16.7)	34 (33.3)
No effect	1 (3.6)	1 (16.6)	2 (5.9)	1 (3.6)	1 (16.6)	2 (5.9)		16 (57.2)	2 (33.4)	18 (53.0)	18 (21.4)	4 (22.2)	**22 (21.6)**
*S. epidermidis* (11)													
Severe effect	0	1 (25.0)	1 (9.1)	0	1 (25.0)	1 (9.1)		0	0	0	0	2 (16.7)	2 (6.0)
Moderate effect	1 (14.3)	0	1 (9.1)	0	0	0		0	0	0	1 (4.7)	0	1 (3.0)
Slight effect	0	0	0	0	0	0		3 (42.9)	1 (25.0)	4 (36.4)	3 (14.3)	1 (8.3)	4 (12.0)
No effect	6 (85.7)	3 (75.0)	9 (81.8)	7 (100)	3 (75.0)	10 (90.9)		4 (57.1)	3 (75.0)	7 (63.6)	17 (81.0)	9 (75.0)	**26 (79.0)**
*S. simulans* (14)													
Severe effect	9 (90.0)	3 (75.0)	12 (85.7)	4 (40.0)	1 (25.0)	5 (35.7)		0	0	0	13 (43.3)	4 (33.4)	17 (40.5)
Moderate effect	1 (10.0)	1 (25.0)	2 (14.3)	6 (60.0)	3 (75.0)	9 (64.3)		2 (20.0)	0	2 (14.3)	9 (30.0)	4 (33.4)	13 (31.0)
Slight effect	0	0	0	0	0	0		5 (50.0)	3 (75.0)	8 (57.2)	5 (16.7)	3 (25.0)	8 (19.0)
No effect	0	0	0	0	0	0		3 (30.0)	1 (25.0)	4 (28.6)	3 (10.0)	1 (8.2)	**4 (9.5)**

^1^Culture preparations = SP–Supernatant; SPK–Supernatant + Proteinase K; CS–Cell suspension.

^2^Number of isolates.

^3^Regulation effect was rated according to the size of inhibition zone around the well (measured in mm) [12] and was classified as exhibiting no effect (≤ 10 mm), a slight effect (11–15 mm), a moderate effect (16–20 mm), and a severe effect (≥ 25 mm) on gene expression.

## Discussion

We demonstrated that bovine-associated NAS downregulate the *S. aureus rnaIII* gene of the strain when sharing the same niche in vitro, an effect that is much more pronounced in *S. chromogenes* and *S. simulans* than it is in *S. epidermidis.* The difference in downregulation between *S. chromogenes* and *S. simulans* on the one hand and *S. epidermidis* on the other hand, is even slightly more pronounced when the in vitro growth inhibition on *S. aureus* is taken into account. As well, we demonstrated that *rnaIII* regulation observed in the liquid assay was comparable with the results from the plate assay, with less effect of *S. epidermidis*, and no differences in effect according to the origin of the NAS isolates (milk versus teat apices). The plate assay also showed that substances produced by the NAS can regulate *rnaIII*, *hla*, and *spa* expression. The effects of the washed NAS cells themselves were less pronounced.

This is the first study that investigated whether the in vitro growth-inhibitory effect of *S. aureus* by bovine NAS also affects the activation of *S. aureus agr* system. Previously it has been suggested that bacteriocins produced by NAS, mainly originating from teat apices, are responsible for growth inhibition of *S. aureus* [16, 27, 39–41]. Some studies also reported the role of bacteriocins as signaling peptides in quorum sensing and bacterial cross talk within microbial communities [42], but none described this mechanism specifically for the NAS species. In our study, total growth inhibition of *S. aureus* by two bovine NAS isolates belonging to *S. chromogenes* (isolate SC29–likely bacteriocin producer, Additional file 1) and *S. simulans* and both originating from teat apices was confirmed, next to partial *S. aureus* inhibition exerted by most of the other isolates. The in vitro growth inhibition pattern, however, did not have a major impact on the growth of *S. aureus* when sharing the niche in the short span (4 h) of the experiments*.* As shown, in a separate small experiment with a subset of NAS isolates, the number of *S. aureus* cells actually remained unchanged within the time frame of the beta-galactosidase liquid assay (4 h) (see Additional file 2). In addition, when the statistical model was corrected by including the effect of growth inhibition, only a slightly change in the LSM of the species effects was observed. Actually, correcting for the inhibitory effect of NAS showed that the downregulation of *rnaIII* would be slightly more pronounced when such effect would not be present. These results suggest that some NAS isolates can affect the activation of *S. aureus agr* system via a mechanism that does not involve growth inhibition.

Despite the well documented literature suggesting the effect of NAS on the regulation of the *agr* system [12, 30–32], our understanding of the crosstalk between bovine-related NAS and *S. aureus* is still limited [33, 43]. In that respect, the use of the laboratory strain 8325–4 has been widely accepted in the animal research field since it was first used [44]. Since, the strain has served as a model for global virulence regulation in *S. aureus* [12, 30, 33]. Here, we present data confirming the capacity of different NAS to suppress *S. aureus rnaIII* expression, a finding that aligns with the results from Mahmmod et al. [33], in which NAS originating from milk and teat apices from dairy cows had the ability of cross-interfering with the *S. aureus agr* quorum sensing system.

Although the majority of the NAS isolates were able to regulate *agr* in our study, it was more pronounced in *S. chromogenes* and *S. simulans* isolates. Nevertheless, the degree of *agr* regulation displayed by the NAS isolates (no effect to severe effect) was not the same, suggesting that the ecological niche the isolates originate from might also be important [12]. However, we report that the *agr* activity of *S. aureus* was equally regulated by isolates originating from milk and TA, which is slightly different from what was reported earlier [33] where NAS isolates originating from TA numerically appear to be more likely to regulate *S. aureus agr*. In our study, differences observed within and between species may be related to the different traits of the NAS isolates. Therefore, further investigations at the strain level must be carried out for a better understanding of their behavior.

It has been demonstrated that the signaling molecules produced by NAS resemble the AIPs of the *S. aureus agr* quorum sensing system [12, 30–32]. Others suggested that AIP-like molecules present in the culture supernatant of NAS might be responsible for downregulating *rnaIII* and *hla* expression and upregulating expression of *spa* [33]. We not only studied the effect of NAS supernatant (SP) on the regulation of *rnaIII*, *hla*, and *spa* but also of supernatant treated with proteinase K (SPK), and cell suspension (CS). Our finding that SP obtained from different NAS regulates the *agr* system of *S. aureus* aligns with previous studies [12, 30, 33]. Canovas et al. [12] reported a considerable suppression across all four *agr* groups of *S. aureus* conferred by the AIP-containing supernatant of the *S. schleiferi* strain 2898 we also used as positive control*.* They also reported that the pure synthetic *S. schleiferi* AIP was able to completely abolish *agr* induction of an *S. aureus* reporter strain. Later, Peng et al. [30] showed that NAS isolated from pigs regulate the *agr* system by competing with the *S. aureus* AIPs for AgrC binding, generally resulting in suppression of the *S. aureus agr*. Although Mahmmod et al. [33] did not identify the exact mechanism involved in the cross-talk between *S. aureus* and NAS originating from milk and TA, they suggested that AIP-like molecules are most likely responsible for the *agr*-suppression caused by their NAS isolates.

The overall regulatory effect of proteinase K treated supernatant (SPK) was not different from the non-treated supernatants (SP). Notwithstanding that we did not isolate and identify which compounds are responsible for such regulatory mechanism, we speculate that NAS produce AIP-like molecules, which are resistant to proteinase K [45], that inhibit the *S. aureus agr* system. Preliminary results of additional tests performed in our lab seem to substantiate that nature (data not shown). These peptides are synthesized in the bacterial cell and transported across the cell membrane by specialized membrane transport proteins [8]. *agr* activity in the bacterial cells will be activated during the late log-phase bacterial growth, when the concentration of the AIP in the medium is high [46]. As the AIP molecule is secreted extracellularly with the higher concentration obtained at late log-phase bacterial growth, we speculate that the concentration of the AIP molecules must be greater in SP and SPK than in the CS, which could partially explain our results. This hypothesis is supported when we consider the results of the plate assay. The use of *lacZ* as a gene reporter in the *S. aureus* strains (Additional file 1) claims to measure the *rnaIII*, *hla*, and *spa* promoter activity under selective pressure of erythromycin [47], a condition to guarantee plasmid stability. Therefore, we consider that growth and production of metabolites by NAS cells were probably reduced in erythromycin-susceptible isolates, affecting the capacity of CS to regulate the virulence genes of *S. aureus*, and potentially (partially) explaining the reduced CS effects*.* This data shown that the *agr* regulatory effect is probably not related to the use of cells (CS), but with metabolites present in the supernatant (SP and SPK); however, further investigations have to be carried out to confirm such hypothesis.

Previous studies identified *S. epidermidis* AIP as a potent suppressor of the *S. aureus agr* system, which might explain the predominance of *S. epidermidis* on the human skin [31]. In contrast, we observed a less pronounced regulatory effect of the *S. epidermidis* isolates against *S. aureus* growth as well as on the regulation of the expression of the *S. aureus agr* system compared with *S. chromogenes* and *S. simulans*. Our data corroborate with the findings of Mahmmod et al. [33], which also reported a variable effect of *S. epidermidis* isolated from milk and teat apex on the regulation of *S. aureus agr* system.

The low number and limited diversity (i.e. three species from two habitats) of the bovine NAS isolates are considered limitations of this study. Future studies should include more species originating from more habitats and could e.g. include NAS from feces [41], a recently described niche yielding NAS in dairy cows. Nevertheless, our findings distinctly show the interactions between *S. aureus* and different NAS originating from milk and TA of dairy cows. They contribute to our understanding of niche competition and colonization between and by staphylococci, although the biological importance of these complex interactions in the bovine mammary gland remains unclear [12, 30, 31, 33, 48]. Such findings are crucial to further advance our understanding of how NAS can play a role in bovine udder health, which might be essentially important to maintain a healthy mammary gland. Accordingly, future studies in animal models (mice and cows) are needed to fully understand these in vitro findings. These studies will also be helpful to confirm (or re-interpret) our results and to address the potential of NAS to target *S. aureus* virulence in the bovine mammary gland.

In conclusion, NAS species isolated from milk and teat apex were able to affect *S. aureus agr* quorum sensing system induction within 4 h. The in vitro growth-inhibitory effect of *S. aureus* by bovine NAS resulted in a slight underestimation of the downregulating effect of NAS on *rnaIII* expression of *S. aureus*. In addition, metabolites produced and secreted into the supernatant by the majority of the bovine NAS isolates, especially by *S. chromogenes* and *S. simulans* isolates, were effective in modulating virulence genes expression as a result of *S. aureus agr* suppression. Interestingly, the habitat of the NAS isolates did not play an important role in the in the results observed. Besides the ecological aspects of *agr* regulation and niche sharing, our findings may open new venues for isolation and identification of potential metabolites produced and secreted by NAS species, their mechanisms of action, and applications as alternative anti-virulence strategies targeting *S. aureus* mastitis treatment and control.

## Supplementary Information

**Additional file 1. Identification of non-aureus staphylococci (NAS) and Staphylococcus aureus isolates and their origin.**

**Additional file 2. β-Galactosidase liquid assay: effect of non-aureus staphylococci (NAS) on growth of Staphylococcus aureus over time. (A)** Growth was monitored as colony forming units (CFUs)/mL. *Staphylococcus aureus rnaIII*::*lacZ* reporter strain SH101F7 was monitored for 4 h growing alone (negative control) in TSB, or in co-culture (1:1) with a set of NAS isolates from *Staphylococcus chromogenes* (*n* = 8), *Staphylococcus epidermidis* (*n* = 6), *Staphylococcus simulans* (*n* = 6), and *Staphylococcus schleiferi* strain 2898 (positive control). **(B)** The NAS isolates were originating from milk (*n* = 11) or teat apices (TA) (*n* = 9). After serial dilution in sterile water, bacterial cultures were plated on TSA with X-gal to differentiate NAS isolates (white colonies) from *S. aureus* (blue colonies). Data presented were an average of two tests in triplicate for each isolate ± standard deviation. Statistical significance was measured using a 1-way ANOVA with a Tukey post-test, *P ≤ 0.05 and ** *P* ≤ 0.001.

## Data Availability

The data on which the conclusions of the manuscript rely are presented in the main paper and additional files.

## Abbreviations

AIP: Auto-inducing peptide
*agr*: Accessory gene regulator
CS: Cell suspension
h: Hours
*hla*: α-Hemolysin
MALDI-Tof: Matrix assisted laser desorption/ionization time-of-fight mass spectrometry
MSA: Mannitol salt agar
no: Number
NAS: Non-*aureus* staphylococci
QS: Quorum sensing
*rnaIII*: Regulatory ribonucleic acid
*S.*: *Staphylococcus*
SP: Culture supernatant
*spa*: Staphylococcal protein A
SPK: Culture supernatant treated with proteinase K
TA: Teat apex
TSB: Tryptic Soy Broth
SCC: Somatic cell count
