# Supplementary material for: Metabolites of bovine-associated non-aureus staphylococci influence expression of Staphylococcus aureus agr-related genes in vitro
Source: Vet Res. 2021 Apr 29;52:62. doi: 10.1186/s13567-021-00933-x (PMC8082617; doi:10.1186/s13567-021-00933-x)
Supplement: Supplementary file 1 — Additional file 1. Identification of non-aureus staphylococci (NAS) and Staphylococcus aureus isolates and their origin. [file 13567_2021_933_MOESM1_ESM.docx]

| Species and isolates | Origin | Reference | | |
| --- | --- | --- | --- | --- |
| NAS |  |  | | |
| *S. chromogenes* |  |  | | |
| SC1 - “IM”^1^ | Bovine milk | [23] | | |
| SC2 | Bovine milk | This study | | |
| SC3 | Bovine milk | This study | | |
| SC4 | Bovine milk | This study | | |
| SC5 | Bovine milk | This study | | |
| SC6 | Bovine milk | This study | | |
| SC7 | Bovine milk | This study | | |
| SC8 | Bovine milk | This study | | |
| SC9 | Bovine milk | This study | | |
| SC10 | Bovine milk | This study | | |
| SC11 | Bovine milk | This study | | |
| SC12 | Bovine milk | This study | | |
| SC13 | Bovine milk | This study | | |
| SC14 | Bovine milk | This study | | |
| SC15 | Bovine milk | This study | | |
| SC16 | Bovine milk | This study | | |
| SC17 | Bovine milk | This study | | |
| SC18 | Bovine milk | This study | | |
| SC19 | Bovine milk | This study | | |
| SC20 | Bovine milk | This study | | |
| SC21 | Bovine milk | This study | | |
| SC22 | Bovine milk | This study | | |
| SC23 | Bovine milk | This study | | |
| SC24 | Bovine milk | This study | | |
| SC25 | Bovine milk | This study | | |
| SC26 | Bovine milk | This study | | |
| SC27 | Bovine milk | This study | | |
| SC28 | Bovine milk | | This study |  |
| SC29 - “TA” | Bovine teat apex | | [27] (C2) |  |
| SC30 | Bovine teat apex | | This study |  |
| SC31 | Bovine teat apex | | This study |  |
| SC32 | Bovine teat apex | | This study |  |
| SC33 | Bovine teat apex | | This study |  |
| SC34 | Bovine teat apex | | This study |  |
| *S. epidermidis* |  | |  |  |
| SE1 | Bovine milk | | This study |  |
| SE2 | Bovine milk | | This study |  |
| SE3 | Bovine milk | | This study |  |
| SE4 | Bovine milk | | This study |  |
| SE5 | Bovine milk | | This study |  |
| SE6 | Bovine milk | | This study |  |
| SE7 | Bovine milk | | This study |  |
| SE8 | Bovine teat apex | | This study |  |
| SE9 | Bovine teat apex | | This study |  |
| SE10 | Bovine teat apex | | This study |  |
| SE11 | Bovine teat apex | | This study |  |
| *S. simulans* |  | |  |  |
| SS1 | Bovine milk | | This study |  |
| SS2 | Bovine milk | | This study |  |
| SS3 | Bovine milk | | This study |  |
| SS4 | Bovine milk | | This study |  |
| SS5 | Bovine milk | | This study |  |
| SS6 | Bovine milk | | This study |  |
| SS7 | Bovine milk | | This study |  |
| SS8 | Bovine milk | | This study |  |
| SS9 | Bovine milk | | This study |  |
| SS10 | Bovine milk | | This study |  |
| SS11 | Bovine teat apex | | This study |  |
| SS12 | Bovine teat apex | | This study |  |
| SS13 | Bovine teat apex | | This study |  |
| SS14 | Bovine teat apex | | This study |  |
| *S. schleiferi* |  | |  |  |
| 2898 | Dog | | [12] |  |
| *S. aureus* |  | |  |  |
| 8325-4 | Wild-type (*agr group I*) | | [44] |  |
| PC322 | 8325-4 *hla::lacZ* Ey*^r^* | | [47] |  |
| PC203 | 8325-4 *spa::lacZ* Ey*^r^* | | [47] |  |
| SH101F7 | 8325-4 *rnaIII::lacZ* Ey*^r^* | | [47] |  |
