# Supplementary figures and images for: Metabolites of bovine-associated non-aureus staphylococci influence expression of Staphylococcus aureus agr-related genes in vitro
Source: Vet Res. 2021 Apr 29;52:62. doi: 10.1186/s13567-021-00933-x (PMC8082617; doi:10.1186/s13567-021-00933-x)

**(A)**


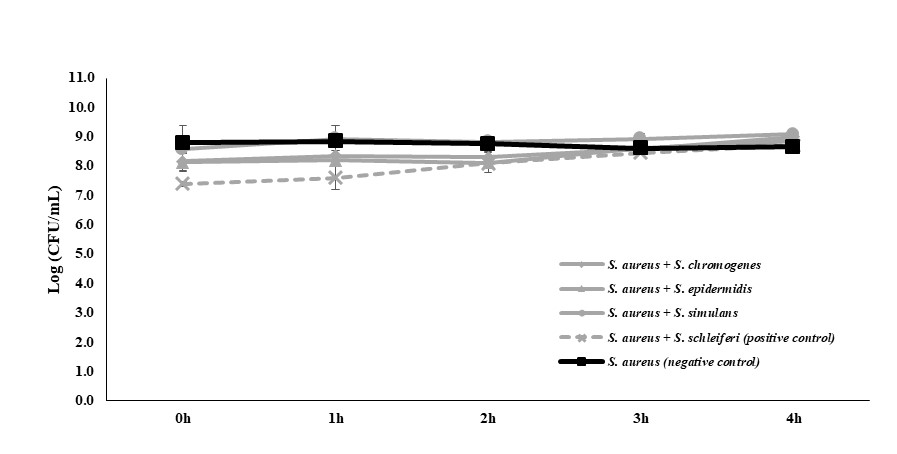


**(B)**


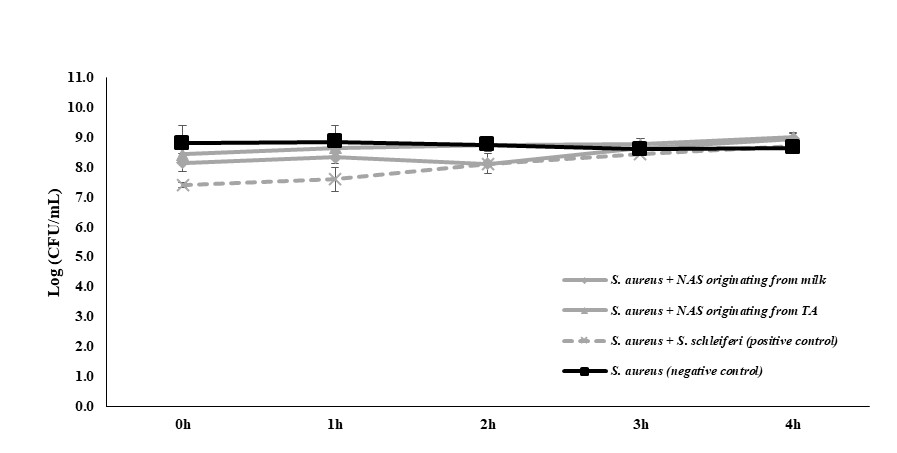

Supplement: Supplementary file 2 — Additional file 2. β-Galactosidase liquid assay: effect of non-aureus staphylococci (NAS) on growth of Staphylococcus aureus over time. (A) Growth was monitored as colony forming units (CFUs)/mL. Staphylococcus aureus rnaIII::lacZ reporter strain SH101F7 was monitored for 4 h growing alone (negative control) in TSB, or in co-culture (1:1) with a set of NAS isolates from Staphylococcus chromogenes (n = 8), Staphylococcus epidermidis (n = 6), Staphylococcus simulans (n = 6), and Staphylococcus schleiferi strain 2898 (positive control). (B) The NAS isolates were originating from milk (n = 11) or teat apices (TA) (n = 9). After serial dilution in sterile water, bacterial cultures were plated on TSA with X-gal to differentiate NAS isolates (white colonies) from S. aureus (blue colonies). Data presented were an average of two tests in triplicate for each isolate ± standard deviation. Statistical significance was measured using a 1-way ANOVA with a Tukey post-test, *P ≤ 0.05 and ** P ≤ 0.001. [file 13567_2021_933_MOESM2_ESM.docx]
